# Supplementary material for: Targeting grasp-related cortical areas for intracortical brain-machine interfaces
Source: Neuroimage Rep. 2026 Jul 10;6(3):100381. doi: 10.1016/j.ynirp.2026.100381 (PMC13380800; doi:10.1016/j.ynirp.2026.100381)
Supplement: Multimedia component 1 [file mmc1.docx]

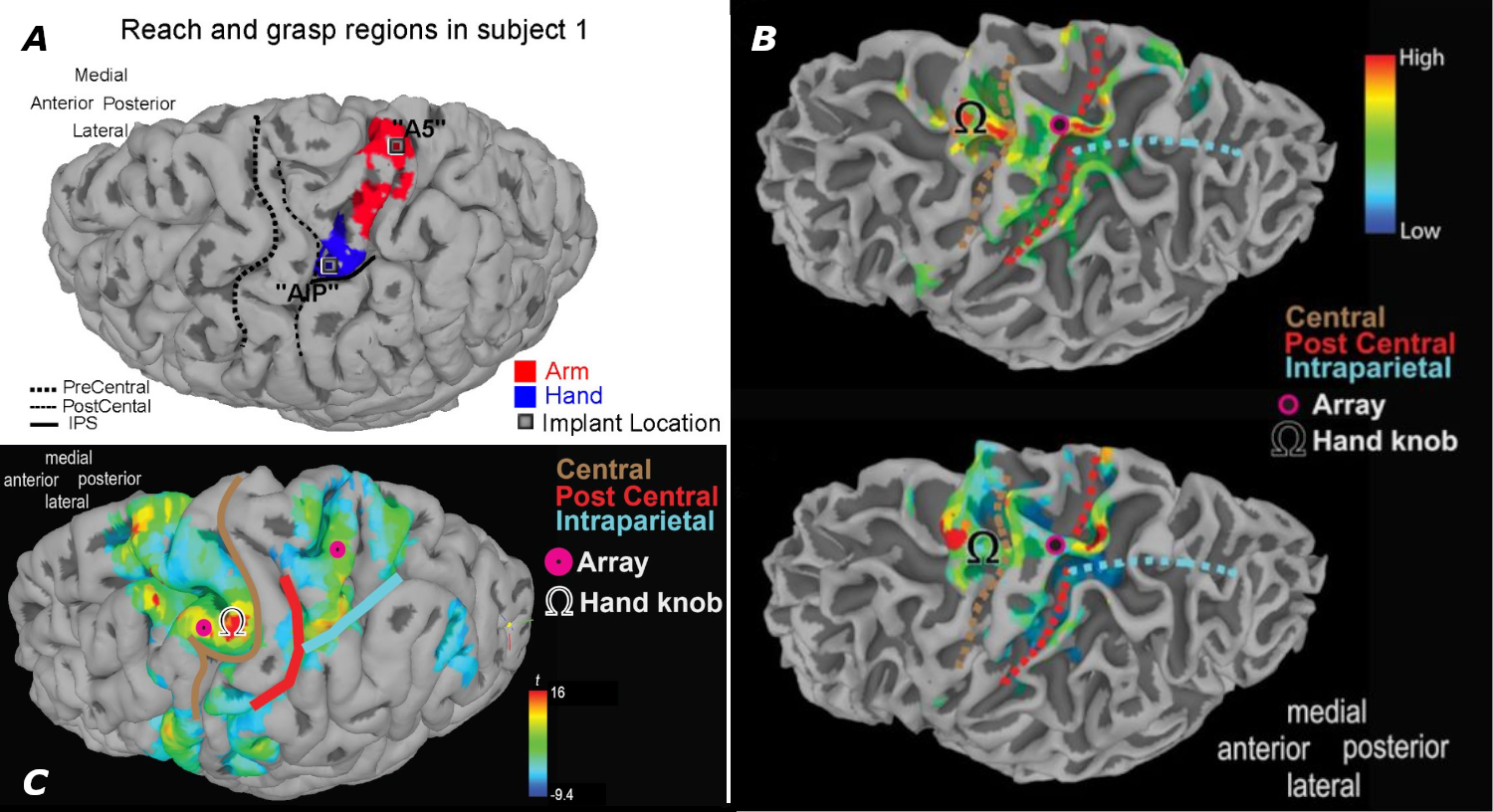


**Supplementary Figure 1 Array implant Locations from other studies targeting grasp/reach related regions** Variability of array placement from A) Aflalo 2015 (Figure 1B), B) Guan 2021 (Figure S1C/D), C) Guan 2022 (Figure S1)


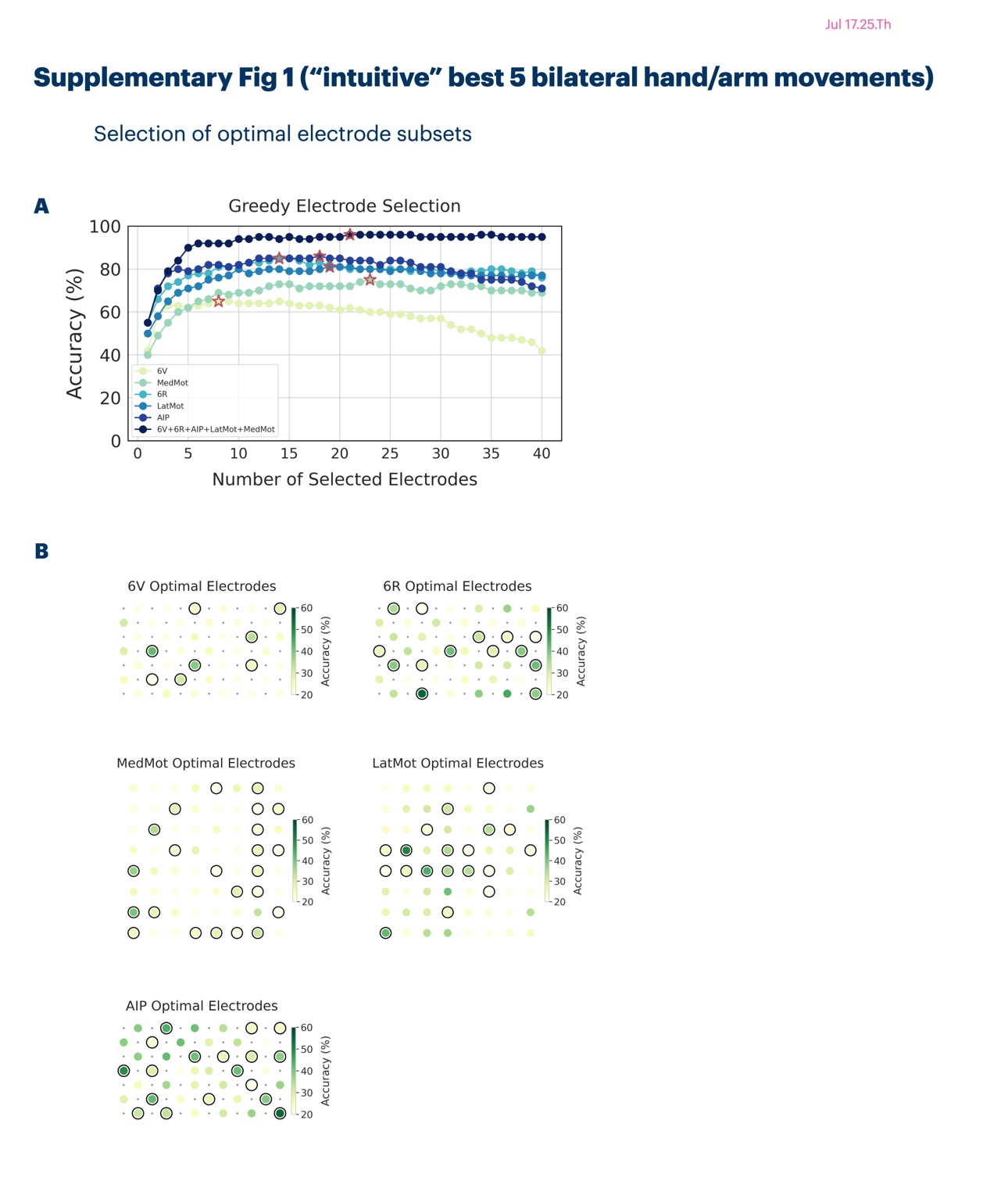


**Supplementary Figure 2 Selection of optimal electrode subsets**

(A) Greedy electrode selection performance. Classification accuracy as a function of the number of electrodes included, using the greedy forward selection procedure (see Methods). For most arrays, performance increases rapidly with the first few electrodes and then plateaus, likely reflecting redundant or overlapping representations across electrodes within the same array. (B). Optimally selected electrodes. Spatial maps of each array showing the subset of electrodes selected for decoding. Highlighted electrodes were included in the final model and used in subsequent population-level analyses.
